# Supplementary material for: Molecular Detection and Characterization of Blastocystis sp. and Enterocytozoon bieneusi in Cattle in Northern Spain
Source: Vet Sci. 2021 Sep 11;8(9):191. doi: 10.3390/vetsci8090191 (PMC8473172; doi:10.3390/vetsci8090191)
Supplement: Supplementary file 1 [file vetsci-08-00191-s001.zip › vetsci-1341255-supplementary.pdf]

Sample_id	Sampling campaign	Sex	Breed	Sampling site	Municipality	County	Production system	*Blastocystis ssu* - PCR	Sanger sequencing	
PTC + 25-1	1	Unknown	Unknown	Unknown	Unknown	Unknown	Unknown	Negative	–	
PTC + • 25-1	1	Unknown	Unknown	Unknown	Unknown	Unknown	Unknown	Dubious	Untypable	–
PTC 1 28-1	1	Unknown	Unknown	Unknown	Unknown	Unknown	Unknown	Negative	–	–
PTC 2 28-1	1	Unknown	Unknown	Unknown	Unknown	Unknown	Unknown	Dubious	Untypable	–
PTC 3 28-1	1	Unknown	Unknown	Unknown	Unknown	Unknown	Unknown	Negative	–	–
PTC 4 28-1	1	Unknown	Unknown	Unknown	Unknown	Unknown	Unknown	Negative	–	–
PTC 5 28-1	1	Unknown	Unknown	Unknown	Unknown	Unknown	Unknown	Negative	–	–
PTC 6 28-1	1	Unknown	Unknown	Unknown	Unknown	Unknown	Unknown	Negative	–	–
PTC 7 28-1	1	Unknown	Unknown	Unknown	Unknown	Unknown	Unknown	Negative	–	–
PTC 8 28-1	1	Unknown	Unknown	Unknown	Unknown	Unknown	Unknown	Negative	–	–
PTC 9 28-1	1	Unknown	Unknown	Unknown	Unknown	Unknown	Unknown	Dubious	Untypable	–
PTC N 28-1	1	Unknown	Unknown	Unknown	Unknown	Unknown	Unknown	Negative	–	–
PTC + 28-1	1	Unknown	Unknown	Unknown	Unknown	Unknown	Unknown	Negative	–	–
PTC + 28 •	1	Unknown	Unknown	Unknown	Unknown	Unknown	Unknown	Dubious	Untypable	–
18210215	1	Female	Holstein-Friesian	Amurrio	Lezama	Ayala	Dairy	Negative	–	–
18210216	1	Female	Holstein-Friesian	Amurrio	Lezama	Ayala	Dairy	Negative	–	–
18210217	1	Female	Holstein-Friesian	Amurrio	Lezama	Ayala	Dairy	Negative	–	–
18210218	1	Female	Holstein-Friesian	Amurrio	Lezama	Ayala	Dairy	Negative	–	–
18210219	1	Female	Holstein-Friesian	Amurrio	Lezama	Ayala	Dairy	Negative	–	–
18210220	1	Female	Holstein-Friesian	Amurrio	Lezama	Ayala	Dairy	Negative	–	–
191741	1	Unknown	Unknown	Abadiño-Zelaieta	Abadiño	Ayala	Unknown	Negative	–	–
191751	1	Female	Pirenaica	Tertanga	Amurrio	Ayala	Beef	Dubious	Untypable	–
191752	1	Female	Pirenaica	Tertanga	Amurrio	Ayala	Beef	Positive	ST10	–
191771	1	Female	Limousin	Zárate	Zuia	Zuia-Gorbeialdea	Beef	Positive	ST5	–
PTB1 29-10 18-1777	1	Female	Holstein-Friesian	Llodio	Llodio	Ayala	Dairy	Dubious	Untypable	–
PTB2 29-10 18-1777	1	Female	Holstein-Friesian	Llodio	Llodio	Ayala	Dairy	Positive	ST5	–
PTB+ 29-10 17-1284	1	Female	Holstein-Friesian	Oleta	Aramaio	Zuia-Gorbeialdea	Dairy	Negative	–	–
Parat1 26-11	1	Unknown	Unknown	Unknown	Unknown	Unknown	Unknown	Negative	–	–
ParatN 26-11	1	Unknown	Unknown	Unknown	Unknown	Unknown	Unknown	Negative	–	–
Parat+26-11	1	Unknown	Unknown	Unknown	Unknown	Unknown	Unknown	Negative	–	–
Par118-12 18/2124	1	Female	Terreña	Guillarte	Kuartango	Añana	Beef	Positive	ST10	–
Par2 18-12 18/2125	1	Female	Pirenaica	Uzkiano	Urkabuztaiz	Zuia-Gorbeialdea	Beef	Dubious	Untypable	–
ParN 18-12 17/1237	1	Female	Mixed breed	Luiando	Ayala	Ayala	Beef	Negative	–	–
Parat+18-12	1	Unknown	Unknown	Unknown	Unknown	Unknown	Unknown	Negative	–	–
PTC125-1	1	Unknown	Astur de montaña	Guillarte	Kuartango	Añana	Beef	Negative	–	–
PTC225-1	1	Unknown	Unknown	Unknown	Unknown	Unknown	Unknown	Negative	–	–
PTC325-1	1	Unknown	Unknown	Unknown	Unknown	Unknown	Unknown	Negative	–	–
PTC425-1	1	Female	Pirenaica	Oiardo	Urkabuztaiz	Zuia-Gorbeialdea	Beef	Negative	–	–
PTC525-1	1	Unknown	Unknown	Unknown	Unknown	Unknown	Unknown	Negative	–	–
PTC625-1	1	Unknown	Unknown	Unknown	Unknown	Unknown	Unknown	Negative	–	–
PTCN25-1	1	Unknown	Unknown	Unknown	Unknown	Unknown	Unknown	Negative	–	–
171237N	1	Female	BOER	Luiando	Ayala	Ayala	Unknown	Negative	–	–
17152830+1	1	Unknown	Unknown	Unknown	Unknown	Unknown	Unknown	Dubious	Untypable	–
18210221	1	Female	Holstein-Friesian	Lezama	Amurrio	Ayala	Dairy	Negative	–	–
18210222	1	Female	Holstein-Friesian	Lezama	Amurrio	Ayala	Dairy	Negative	–	–
23	1	Female	Holstein-Friesian	Lezama	Amurrio	Ayala	Dairy	Negative	–	–
24	1	Female	Holstein-Friesian	Lezama	Amurrio	Ayala	Dairy	Negative	–	–
25	1	Female	Holstein-Friesian	Lezama	Amurrio	Ayala	Dairy	Negative	–	–
26	1	Female	Holstein-Friesian	Lezama	Amurrio	Ayala	Dairy	Dubious	Untypable	–
27	1	Female	Holstein-Friesian	Lezama	Amurrio	Ayala	Dairy	Negative	–	–
28	1	Female	Holstein-Friesian	Lezama	Amurrio	Ayala	Dairy	Negative	–	–
29	1	Female	Holstein-Friesian	Lezama	Amurrio	Ayala	Dairy	Negative	–	–
30	1	Female	Holstein-Friesian	Lezama	Amurrio	Ayala	Dairy	Dubious	Untypable	–
31	1	Female	Holstein-Friesian	Lezama	Amurrio	Ayala	Dairy	Negative	–	–
32	1	Female	Holstein-Friesian	Lezama	Amurrio	Ayala	Dairy	Negative	–	–
33	1	Female	Holstein-Friesian	Lezama	Amurrio	Ayala	Dairy	Negative	–	–
34	1	Female	Holstein-Friesian	Lezama	Amurrio	Ayala	Dairy	Negative	–	–
35	1	Female	Holstein-Friesian	Lezama	Amurrio	Ayala	Dairy	Negative	–	–
36	1	Female	Holstein-Friesian	Lezama	Amurrio	Ayala	Dairy	Negative	–	–
37	1	Female	Holstein-Friesian	Lezama	Amurrio	Ayala	Dairy	Negative	–	–
38	1	Female	Holstein-Friesian	Lezama	Amurrio	Ayala	Dairy	Dubious	Untypable	–
39	1	Female	Holstein-Friesian	Lezama	Amurrio	Ayala	Dairy	Positive	ST10	–
18210240	1	Female	Holstein-Friesian	Lezama	Amurrio	Ayala	Dairy	Dubious	Untypable	–
PTC17/1237N 31-1	1	Female	BOER	Luiando	Ayala	Ayala	Unknown	Negative	–	–
PTC+18/1528 31-1	1	Female	Astur de montaña	Subijana-Morillas	Ribera alta	Añana	Beef	Dubious	Untypable	–
18210241	1	Female	Holstein-Friesian	Lezama	Amurrio	Ayala	Dairy	Dubious	Untypable	–
18210242	1	Female	Holstein-Friesian	Lezama	Amurrio	Ayala	Dairy	Dubious	Untypable	–
18210243	1	Female	Holstein-Friesian	Lezama	Amurrio	Ayala	Dairy	Dubious	Untypable	–
18210244	1	Female	Holstein-Friesian	Lezama	Amurrio	Ayala	Dairy	Positive	ST10	–
18210245	1	Female	Holstein-Friesian	Lezama	Amurrio	Ayala	Dairy	Dubious	Untypable	–
18210246	1	Female	Holstein-Friesian	Lezama	Amurrio	Ayala	Dairy	Dubious	Untypable	–
18210247	1	Female	Holstein-Friesian	Lezama	Amurrio	Ayala	Dairy	Negative	–	–
18210248	1	Female	Holstein-Friesian	Lezama	Amurrio	Ayala	Dairy	Dubious	Untypable	–
18210249	1	Female	Holstein-Friesian	Lezama	Amurrio	Ayala	Dairy	Dubious	Untypable	–
18210250	1	Female	Holstein-Friesian	Lezama	Amurrio	Ayala	Dairy	Negative	–	–
18210251	1	Female	Holstein-Friesian	Lezama	Amurrio	Ayala	Dairy	Negative	–	–
18210252	1	Female	Holstein-Friesian	Lezama	Amurrio	Ayala	Dairy	Negative	–	–
18210253	1	Female	Holstein-Friesian	Lezama	Amurrio	Ayala	Dairy	Negative	–	–
18210254	1	Female	Holstein-Friesian	Lezama	Amurrio	Ayala	Dairy	Negative	–	–
18210255	1	Female	Holstein-Friesian	Lezama	Amurrio	Ayala	Dairy	Negative	–	–
18210256	1	Female	Holstein-Friesian	Lezama	Amurrio	Ayala	Dairy	Negative	–	–
18210257	1	Female	Holstein-Friesian	Lezama	Amurrio	Ayala	Dairy	Negative	–	–
18210258	1	Female	Holstein-Friesian	Lezama	Amurrio	Ayala	Dairy	Negative	–	–

18210259	1	Female	Holstein-Friesian	Lezama	Amurrio	Ayala	Dairy	Negative	–	–
18210260	1	Female	Holstein-Friesian	Lezama	Amurrio	Ayala	Dairy	Negative	–	–
61	1	Female	Holstein-Friesian	Lezama	Amurrio	Ayala	Dairy	Negative	–	–
62	1	Female	Holstein-Friesian	Lezama	Amurrio	Ayala	Dairy	Negative	–	–
63	1	Female	Holstein-Friesian	Lezama	Amurrio	Ayala	Dairy	Negative	–	–
64	1	Female	Holstein-Friesian	Lezama	Amurrio	Ayala	Dairy	Negative	–	–
65	1	Female	Holstein-Friesian	Lezama	Amurrio	Ayala	Dairy	Negative	–	–
18210276	1	Female	Holstein-Friesian	Lezama	Amurrio	Ayala	Dairy	**Positive**	**ST10**	–
66	1	Female	Holstein-Friesian	Lezama	Amurrio	Ayala	Dairy	Negative	–	–
67	1	Female	Holstein-Friesian	Lezama	Amurrio	Ayala	Dairy	Negative	–	–
68	1	Female	Holstein-Friesian	Lezama	Amurrio	Ayala	Dairy	Negative	–	–
69	1	Female	Holstein-Friesian	Lezama	Amurrio	Ayala	Dairy	Negative	–	–
70	1	Female	Holstein-Friesian	Lezama	Amurrio	Ayala	Dairy	Negative	–	–
71	1	Female	Holstein-Friesian	Lezama	Amurrio	Ayala	Dairy	Negative	–	–
72	1	Female	Holstein-Friesian	Lezama	Amurrio	Ayala	Dairy	Negative	–	–
73	1	Female	Holstein-Friesian	Lezama	Amurrio	Ayala	Dairy	Negative	–	–
74	1	Female	Holstein-Friesian	Lezama	Amurrio	Ayala	Dairy	Negative	–	–
75	1	Female	Holstein-Friesian	Lezama	Amurrio	Ayala	Dairy	Negative	–	–
192131	1	Female	Pirenaica	Arexola	Aramaio	Zuia-Gorbeialdea	Beef	Negative	–	–
192132	1	Female	Pirenaica	Arexola	Aramaio	Zuia-Gorbeialdea	Beef	**Positive**	**ST10**	–
191901	1	Female	Mixed breed	Aperregi	Zuia	Zuia-Gorbeialdea	Beef	**Positive**	**ST10**	–
191902	1	Female	Mixed breed	Aperregi	Zuia	Zuia-Gorbeialdea	Beef	**Positive**	**ST5**	–
192541	1	Female	Astur de montaña	Subijana-Morillas	Ribera alta	Añana	Beef	**Dubious**	Untypable	–
192542	1	Female	Astur de montaña	Subijana-Morillas	Ribera alta	Añana	Beef	**Positive**	**ST5**	–
192543	1	Female	Astur de montaña	Subijana-Morillas	Ribera alta	Añana	Beef	**Positive**	**ST5**	–
192591	1	Female	Pirenaica	Abezia	Urkabuztaiz	Zuia-Gorbeialdea	Beef	**Dubious**	Untypable	–
192741	1	Female	Salers	Ozaeta	Barrundia	Llanada Alavesa	Beef	**Positive**	**ST10**	–
192751	1	Female	Astur de montaña	Subijana-Morillas	Ribera alta	Añana	Beef	**Dubious**	Untypable	–
PTCN12-2	1	Unknown	Unknown	Unknown	Unknown	Unknown	Unknown	Negative	–	–
PTC+12-2	1	Unknown	Unknown	Unknown	Unknown	Unknown	Unknown	**Dubious**	Untypable	–
PTC119387	1	Female	Pirenaica	Oiardo	Urkabuztaiz	Zuia-Gorbeialdea	Beef	Negative	–	–
219387	1	Female	Pirenaica	Oiardo	Urkabuztaiz	Zuia-Gorbeialdea	Beef	**Dubious**	Untypable	–
319387	1	Female	Pirenaica	Oiardo	Urkabuztaiz	Zuia-Gorbeialdea	Beef	**Dubious**	Untypable	–
419387	1	Female	Pirenaica	Oiardo	Urkabuztaiz	Zuia-Gorbeialdea	Beef	**Dubious**	Untypable	–
PTC113-3	1	Unknown	Unknown	Unknown	Unknown	Unknown	Unknown	**Dubious**	Untypable	–
PTC213-3	1	Unknown	Unknown	Unknown	Unknown	Unknown	Unknown	**Positive**	**ST5**	–
PTC313-3	1	Unknown	Unknown	Unknown	Unknown	Unknown	Unknown	**Positive**	**ST10**	–
PTC14-4	1	Unknown	Unknown	Unknown	Unknown	Unknown	Unknown	Negative	–	–
PTC24-4	1	Unknown	Unknown	Unknown	Unknown	Unknown	Unknown	Negative	–	–
PTC34-4	1	Unknown	Unknown	Unknown	Unknown	Unknown	Unknown	**Dubious**	Untypable	–
PTC44-4	1	Unknown	Unknown	Unknown	Unknown	Unknown	Unknown	**Dubious**	Untypable	–
PTC54-4	1	Unknown	Unknown	Unknown	Unknown	Unknown	Unknown	Negative	–	–
PTC64-4	1	Unknown	Unknown	Unknown	Unknown	Unknown	Unknown	**Dubious**	Untypable	–
PTC74-4	1	Unknown	Unknown	Unknown	Unknown	Unknown	Unknown	**Dubious**	Untypable	–
PTC84-4	1	Unknown	Unknown	Unknown	Unknown	Unknown	Unknown	**Dubious**	Untypable	–
PTC121-3	1	Unknown	Unknown	Unknown	Unknown	Unknown	Unknown	**Dubious**	Untypable	–
PTC221-3	1	Unknown	Unknown	Unknown	Unknown	Unknown	Unknown	**Positive**	**ST5**	–
PTC321-3	1	Unknown	Unknown	Unknown	Unknown	Unknown	Unknown	Negative	–	–
PTC421-3	1	Unknown	Unknown	Unknown	Unknown	Unknown	Unknown	Negative	–	–
PTC521-3	1	Unknown	Unknown	Unknown	Unknown	Unknown	Unknown	Negative	–	–
PTC621-3	1	Unknown	Unknown	Unknown	Unknown	Unknown	Unknown	Negative	–	–
PTC721-3	1	Unknown	Unknown	Unknown	Unknown	Unknown	Unknown	Negative	–	–
PTC821-3	1	Unknown	Unknown	Unknown	Unknown	Unknown	Unknown	Negative	–	–
PTC921-3	1	Unknown	Unknown	Unknown	Unknown	Unknown	Unknown	Negative	–	–
PTC1021-3	1	Unknown	Unknown	Unknown	Unknown	Unknown	Unknown	Negative	–	–
PTC1121-3	1	Unknown	Unknown	Unknown	Unknown	Unknown	Unknown	Negative	–	–
PTC1221-3	1	Unknown	Unknown	Unknown	Unknown	Unknown	Unknown	Negative	–	–
PTC1321-3	1	Unknown	Unknown	Unknown	Unknown	Unknown	Unknown	**Positive**	**ST5**	–
PTC1421-3	1	Unknown	Unknown	Unknown	Unknown	Unknown	Unknown	Negative	–	–
PTC115-4	1	Unknown	Unknown	Unknown	Unknown	Unknown	Unknown	Negative	–	–
PTC215-4	1	Unknown	Unknown	Unknown	Unknown	Unknown	Unknown	**Dubious**	Untypable	–
PTC315-4	1	Unknown	Unknown	Unknown	Unknown	Unknown	Unknown	**Dubious**	Untypable	–
PTC415-4	1	Unknown	Unknown	Unknown	Unknown	Unknown	Unknown	**Positive**	**ST10**	–
PTC515-4	1	Unknown	Unknown	Unknown	Unknown	Unknown	Unknown	**Positive**	**ST10**	–
PTC615-4	1	Unknown	Unknown	Unknown	Unknown	Unknown	Unknown	**Dubious**	Untypable	–
PTC715-4	1	Unknown	Unknown	Unknown	Unknown	Unknown	Unknown	**Dubious**	Untypable	–
PTC815-4	1	Unknown	Unknown	Unknown	Unknown	Unknown	Unknown	Negative	–	–
PTC915-4	1	Unknown	Unknown	Unknown	Unknown	Unknown	Unknown	**Dubious**	Untypable	–
PTC1015-4	1	Unknown	Unknown	Unknown	Unknown	Unknown	Unknown	**Positive**	**ST10**	–
PTC1115-4	1	Unknown	Unknown	Unknown	Unknown	Unknown	Unknown	**Dubious**	Untypable	–
PTC1215-4	1	Unknown	Unknown	Unknown	Unknown	Unknown	Unknown	Negative	–	–
PTC1315-4	1	Unknown	Unknown	Unknown	Unknown	Unknown	Unknown	**Dubious**	Untypable	–
PTC1415-4	1	Unknown	Unknown	Unknown	Unknown	Unknown	Unknown	**Dubious**	Untypable	–
PTC1515-4	1	Unknown	Unknown	Unknown	Unknown	Unknown	Unknown	**Dubious**	Untypable	–
PTC1615-4	1	Unknown	Unknown	Unknown	Unknown	Unknown	Unknown	**Dubious**	Untypable	ST3
PTC1715-4	1	Unknown	Unknown	Unknown	Unknown	Unknown	Unknown	**Positive**	**ST5**	–
PTC1815-4	1	Unknown	Unknown	Unknown	Unknown	Unknown	Unknown	**Positive**	**ST5**	–
PTC18-5	1	Unknown	Unknown	Unknown	Unknown	Unknown	Unknown	Negative	–	–
PTC28-5	1	Unknown	Unknown	Unknown	Unknown	Unknown	Unknown	**Positive**	**ST14**	–
PTC38-5	1	Unknown	Unknown	Unknown	Unknown	Unknown	Unknown	**Positive**	**ST10**	–
PTC48-5	1	Unknown	Unknown	Unknown	Unknown	Unknown	Unknown	**Positive**	**ST5**	–
PTC58-5	1	Unknown	Unknown	Unknown	Unknown	Unknown	Unknown	Negative	–	–
PTC68-5	1	Unknown	Unknown	Unknown	Unknown	Unknown	Unknown	**Dubious**	Untypable	–
PTC78-5	1	Unknown	Unknown	Unknown	Unknown	Unknown	Unknown	**Dubious**	Untypable	–
PTC88-5	1	Unknown	Unknown	Unknown	Unknown	Unknown	Unknown	Negative	–	–

PTC98-5	1	Unknown	Unknown	Unknown	Unknown	Unknown	Unknown	Negative	–	–
PTC108-5	1	Unknown	Unknown	Unknown	Unknown	Unknown	Unknown	Positive	ST10	–
PTC118-5	1	Unknown	Unknown	Unknown	Unknown	Unknown	Unknown	Positive	ST10	–
PTC128-5	1	Unknown	Unknown	Unknown	Unknown	Unknown	Unknown	Dubious	Untypable	–
PTC138-5	1	Unknown	Unknown	Unknown	Unknown	Unknown	Unknown	Dubious	Untypable	–
PTC148-5	1	Unknown	Unknown	Unknown	Unknown	Unknown	Unknown	Dubious	Untypable	–
PTC158-5	1	Unknown	Unknown	Unknown	Unknown	Unknown	Unknown	Positive	ST5	–
PTBC116-5	1	Unknown	Unknown	Unknown	Unknown	Unknown	Unknown	Negative	–	–
PTBC216-5	1	Unknown	Unknown	Unknown	Unknown	Unknown	Unknown	Dubious	Untypable	–
PTBC316-5	1	Unknown	Unknown	Unknown	Unknown	Unknown	Unknown	Dubious	Untypable	–
PTBC4	1	Unknown	Unknown	Unknown	Unknown	Unknown	Unknown	Dubious	Untypable	–
PTB 1	1	Unknown	Unknown	Unknown	Unknown	Unknown	Unknown	Negative	–	–
PTB 3	1	Unknown	Unknown	Unknown	Unknown	Unknown	Unknown	Negative	–	–
PTB 19/1726 23.7	1	Unknown	Unknown	Unknown	Unknown	Unknown	Unknown	Negative	–	–
PTB 19/1727 23.7	1	Unknown	Unknown	Unknown	Unknown	Unknown	Unknown	Negative	–	–
PTB 19/1728 23.7	1	Unknown	Unknown	Unknown	Unknown	Unknown	Unknown	Negative	–	–
PTB 19/1729 23.7	1	Unknown	Unknown	Unknown	Unknown	Unknown	Unknown	Negative	–	–
PTB 19/1730 23.7	1	Unknown	Unknown	Unknown	Unknown	Unknown	Unknown	Negative	–	–
PTB 19/1686 1	1	Female	Holstein-Friesian	Ollabarre	Iruña de Oca	Añana	Dairy	Negative	–	–
PTB 19/1707 1	1	Female	Holstein-Friesian	Uzkiano	Urkabuztaiz	Zuia-Gorbeialdea	Dairy	Negative	–	–
PTB 19/1707 2	1	Female	Holstein-Friesian	Uzkiano	Urkabuztaiz	Zuia-Gorbeialdea	Dairy	Positive	ST5	–
PTB 19/1707 5	1	Female	Holstein-Friesian	Uzkiano	Urkabuztaiz	Zuia-Gorbeialdea	Dairy	Positive	ST10	–
PTB 19/1707 7	1	Female	Holstein-Friesian	Uzkiano	Urkabuztaiz	Zuia-Gorbeialdea	Dairy	Positive	ST5	–
PTB 1 29.10	1	Unknown	Unknown	Unknown	Unknown	Unknown	Unknown	Negative	–	–
PTB 2 29.10	1	Unknown	Unknown	Unknown	Unknown	Unknown	Unknown	Positive	ST5	–
PTB 3 29.10	1	Unknown	Unknown	Unknown	Unknown	Unknown	Unknown	Dubious	Untypable	–
PTB 4 29.10	1	Unknown	Unknown	Unknown	Unknown	Unknown	Unknown	Negative	–	–
PTB 5 29.10	1	Unknown	Unknown	Unknown	Unknown	Unknown	Unknown	Positive	ST5	–
PTB 19/1707 1	1	Female	Holstein-Friesian	Uzkiano	Urkabuztaiz	Zuia-Gorbeialdea	Dairy	Negative	–	–
PTB 19/1707 2	1	Female	Holstein-Friesian	Uzkiano	Urkabuztaiz	Zuia-Gorbeialdea	Dairy	Negative	–	–
PTB 19/1707 3	1	Female	Holstein-Friesian	Uzkiano	Urkabuztaiz	Zuia-Gorbeialdea	Dairy	Positive	ST10	–
PTB 19/1707 4	1	Female	Holstein-Friesian	Uzkiano	Urkabuztaiz	Zuia-Gorbeialdea	Dairy	Negative	–	–
PTB 19/1707 5	1	Female	Holstein-Friesian	Uzkiano	Urkabuztaiz	Zuia-Gorbeialdea	Dairy	Positive	ST10	–
PTB 19/1707 6	1	Female	Holstein-Friesian	Uzkiano	Urkabuztaiz	Zuia-Gorbeialdea	Dairy	Dubious	Untypable	–
PTB 19/1707 7	1	Female	Holstein-Friesian	Uzkiano	Urkabuztaiz	Zuia-Gorbeialdea	Dairy	Positive	ST5	–
1871_1	1	Female	Holstein-Friesian	Lezama	Amurrio	Ayala	Dairy	Negative	–	–
1871_2	1	Female	Holstein-Friesian	Lezama	Amurrio	Ayala	Dairy	Negative	–	–
1871_3	1	Female	Holstein-Friesian	Lezama	Amurrio	Ayala	Dairy	Negative	–	–
1871_4	1	Female	Holstein-Friesian	Lezama	Amurrio	Ayala	Dairy	Negative	–	–
1871_5	1	Female	Holstein-Friesian	Lezama	Amurrio	Ayala	Dairy	Negative	–	–
1871_6	1	Female	Holstein-Friesian	Lezama	Amurrio	Ayala	Dairy	Negative	–	–
1871_7	1	Female	Holstein-Friesian	Lezama	Amurrio	Ayala	Dairy	Negative	–	–
1871_8	1	Female	Holstein-Friesian	Lezama	Amurrio	Ayala	Dairy	Negative	–	–
1871_9	1	Female	Holstein-Friesian	Lezama	Amurrio	Ayala	Dairy	Negative	–	–
1895_1	1	Female	Holstein-Friesian	Lukiano	Zuia	Zuia-Gorbeialdea	Dairy	Negative	–	–
1895_2	1	Female	Holstein-Friesian	Lukiano	Zuia	Zuia-Gorbeialdea	Dairy	Negative	–	–
1895_3	1	Female	Holstein-Friesian	Lukiano	Zuia	Zuia-Gorbeialdea	Dairy	Negative	–	–
1895_4	1	Female	Holstein-Friesian	Lukiano	Zuia	Zuia-Gorbeialdea	Dairy	Negative	–	–
1895_5	1	Female	Holstein-Friesian	Lukiano	Zuia	Zuia-Gorbeialdea	Dairy	Negative	–	–
1895_6	1	Female	Holstein-Friesian	Lukiano	Zuia	Zuia-Gorbeialdea	Dairy	Negative	–	–
1895_7	1	Female	Holstein-Friesian	Lukiano	Zuia	Zuia-Gorbeialdea	Dairy	Negative	–	–
1895_8	1	Female	Holstein-Friesian	Lukiano	Zuia	Zuia-Gorbeialdea	Dairy	Negative	–	–
1895_9	1	Female	Holstein-Friesian	Lukiano	Zuia	Zuia-Gorbeialdea	Dairy	Negative	–	–
1895_10	1	Female	Holstein-Friesian	Lukiano	Zuia	Zuia-Gorbeialdea	Dairy	Negative	–	–
1895_11	1	Female	Holstein-Friesian	Lukiano	Zuia	Zuia-Gorbeialdea	Dairy	Negative	–	–
1895_12	1	Female	Holstein-Friesian	Lukiano	Zuia	Zuia-Gorbeialdea	Dairy	Negative	–	–
1895_13	1	Female	Holstein-Friesian	Lukiano	Zuia	Zuia-Gorbeialdea	Dairy	Negative	–	–
1895_14	1	Female	Holstein-Friesian	Lukiano	Zuia	Zuia-Gorbeialdea	Dairy	Negative	–	–
20-1009/1	2	Male	Pirenaica	Sojo	Ayala	Ayala	Beef	Negative	–	–
20-1009/2	2	Female	Mixed breed	Sojo	Ayala	Ayala	Beef	Negative	–	–
20-1009/3	2	Female	Pirenaica	Sojo	Ayala	Ayala	Beef	Negative	–	–
20-1009/4	2	Female	Limousin	Sojo	Ayala	Ayala	Beef	Negative	–	–
20-1009/5	2	Female	Pirenaica	Sojo	Ayala	Ayala	Beef	Negative	–	–
20-1009/6	2	Female	Pirenaica	Sojo	Ayala	Ayala	Beef	Negative	–	–
20-1015/1	2	Female	Holstein-Friesian	Goian	Legutio	Zuia-Gorbeialdea	Dairy	Negative	–	–
20-1026/1	2	Female	Limousin	Goiuri	Urkabuztaiz	Zuia-Gorbeialdea	Beef	Negative	–	–
20-1026/2	2	Female	Limousin	Goiuri	Urkabuztaiz	Zuia-Gorbeialdea	Beef	Negative	–	–
20-1026/3	2	Female	Limousin	Goiuri	Urkabuztaiz	Zuia-Gorbeialdea	Beef	Negative	–	–
20-1026/4	2	Female	Limousin	Goiuri	Urkabuztaiz	Zuia-Gorbeialdea	Beef	Negative	–	–
20-1026/5	2	Female	Limousin	Goiuri	Urkabuztaiz	Zuia-Gorbeialdea	Beef	Negative	–	–
20-1026/6	2	Female	Limousin	Goiuri	Urkabuztaiz	Zuia-Gorbeialdea	Beef	Negative	–	–
20-1026/7	2	Female	Limousin	Goiuri	Urkabuztaiz	Zuia-Gorbeialdea	Beef	Negative	–	–
20-1026/8	2	Female	Limousin	Goiuri	Urkabuztaiz	Zuia-Gorbeialdea	Beef	Negative	–	–
20-1026/31	2	Female	Limousin	Goiuri	Urkabuztaiz	Zuia-Gorbeialdea	Beef	Negative	–	–
20-1004-1	2	Female	Holstein-Friesian	Oiardo	Urkabuztaiz	Zuia-Gorbeialdea	Dairy	Dubious	Untypable	–
20-1004-2	2	Female	Holstein-Friesian	Oiardo	Urkabuztaiz	Zuia-Gorbeialdea	Dairy	Dubious	Untypable	–
20-1004-3	2	Female	Holstein-Friesian	Oiardo	Urkabuztaiz	Zuia-Gorbeialdea	Dairy	Dubious	Untypable	–
20-1004-4	2	Female	Holstein-Friesian	Oiardo	Urkabuztaiz	Zuia-Gorbeialdea	Dairy	Negative	–	–
20-1004-5	2	Female	Holstein-Friesian	Oiardo	Urkabuztaiz	Zuia-Gorbeialdea	Dairy	Dubious	Untypable	–
20-1004-6	2	Female	Holstein-Friesian	Oiardo	Urkabuztaiz	Zuia-Gorbeialdea	Dairy	Dubious	Untypable	–
20-1004-11	2	Female	Holstein-Friesian	Oiardo	Urkabuztaiz	Zuia-Gorbeialdea	Dairy	Dubious	Untypable	–
20-1004-12	2	Female	Holstein-Friesian	Oiardo	Urkabuztaiz	Zuia-Gorbeialdea	Dairy	Negative	–	–
20-1004-13	2	Female	Holstein-Friesian	Oiardo	Urkabuztaiz	Zuia-Gorbeialdea	Dairy	Negative	–	–
20-1004-14	2	Female	Holstein-Friesian	Oiardo	Urkabuztaiz	Zuia-Gorbeialdea	Dairy	Negative	–	–
20-1004-15	2	Female	Holstein-Friesian	Oiardo	Urkabuztaiz	Zuia-Gorbeialdea	Dairy	Negative	–	–

20-1004-16	2	Female	Holstein-Friesian	Oiardo	Urkabuztaiz	Zuia-Gorbeialdea	Dairy	Negative	–	–
20-1004-17	2	Female	Holstein-Friesian	Oiardo	Urkabuztaiz	Zuia-Gorbeialdea	Dairy	Negative	–	–
20-1004-18	2	Female	Holstein-Friesian	Oiardo	Urkabuztaiz	Zuia-Gorbeialdea	Dairy	Negative	–	–
20-1004-20	2	Female	Holstein-Friesian	Oiardo	Urkabuztaiz	Zuia-Gorbeialdea	Dairy	Negative	–	–
20-1004-22	2	Female	Holstein-Friesian	Oiardo	Urkabuztaiz	Zuia-Gorbeialdea	Dairy	Negative	–	–
20-1004-28	2	Female	Holstein-Friesian	Oiardo	Urkabuztaiz	Zuia-Gorbeialdea	Dairy	Negative	–	–
20-1004-31	2	Female	Holstein-Friesian	Oiardo	Urkabuztaiz	Zuia-Gorbeialdea	Dairy	Dubious	Untypable	–
20-1004-32	2	Female	Holstein-Friesian	Oiardo	Urkabuztaiz	Zuia-Gorbeialdea	Dairy	Dubious	Untypable	–
20-1004-33	2	Female	Holstein-Friesian	Oiardo	Urkabuztaiz	Zuia-Gorbeialdea	Dairy	Negative	–	–
20-1004-34	2	Female	Holstein-Friesian	Oiardo	Urkabuztaiz	Zuia-Gorbeialdea	Dairy	Negative	–	–
20-1004-35	2	Female	Holstein-Friesian	Oiardo	Urkabuztaiz	Zuia-Gorbeialdea	Dairy	Negative	–	–
20-1004-36	2	Female	Holstein-Friesian	Oiardo	Urkabuztaiz	Zuia-Gorbeialdea	Dairy	Negative	–	–
20-1004-37	2	Female	Holstein-Friesian	Oiardo	Urkabuztaiz	Zuia-Gorbeialdea	Dairy	Negative	–	–
20-1004-38	2	Female	Holstein-Friesian	Oiardo	Urkabuztaiz	Zuia-Gorbeialdea	Dairy	Dubious	Untypable	–
20-1004-41	2	Female	Holstein-Friesian	Oiardo	Urkabuztaiz	Zuia-Gorbeialdea	Dairy	Negative	–	–
20-1004-42	2	Female	Holstein-Friesian	Oiardo	Urkabuztaiz	Zuia-Gorbeialdea	Dairy	Negative	–	–
20-1004-45	2	Female	Holstein-Friesian	Oiardo	Urkabuztaiz	Zuia-Gorbeialdea	Dairy	Negative	–	–
20-1004-46	2	Female	Holstein-Friesian	Oiardo	Urkabuztaiz	Zuia-Gorbeialdea	Dairy	Negative	–	–
20-1004-47	2	Female	Holstein-Friesian	Oiardo	Urkabuztaiz	Zuia-Gorbeialdea	Dairy	Negative	–	–
20-1004-48	2	Female	Holstein-Friesian	Oiardo	Urkabuztaiz	Zuia-Gorbeialdea	Dairy	Negative	–	–
20-1004-49	2	Female	Holstein-Friesian	Oiardo	Urkabuztaiz	Zuia-Gorbeialdea	Dairy	Dubious	Untypable	–
20-1004-50	2	Female	Holstein-Friesian	Oiardo	Urkabuztaiz	Zuia-Gorbeialdea	Dairy	Negative	–	–
20-1004-51	2	Female	Holstein-Friesian	Oiardo	Urkabuztaiz	Zuia-Gorbeialdea	Dairy	Negative	–	–
20-1004-52	2	Female	Holstein-Friesian	Oiardo	Urkabuztaiz	Zuia-Gorbeialdea	Dairy	Negative	–	–
20-1004-53	2	Female	Holstein-Friesian	Oiardo	Urkabuztaiz	Zuia-Gorbeialdea	Dairy	Negative	–	–
20-1004-54	2	Female	Holstein-Friesian	Oiardo	Urkabuztaiz	Zuia-Gorbeialdea	Dairy	Negative	–	–
20-1004-55	2	Female	Holstein-Friesian	Oiardo	Urkabuztaiz	Zuia-Gorbeialdea	Dairy	Negative	–	–
20-1004-56	2	Female	Holstein-Friesian	Oiardo	Urkabuztaiz	Zuia-Gorbeialdea	Dairy	Negative	–	–
20-1004-57	2	Female	Holstein-Friesian	Oiardo	Urkabuztaiz	Zuia-Gorbeialdea	Dairy	Dubious	Untypable	–
20-1004-58	2	Female	Holstein-Friesian	Oiardo	Urkabuztaiz	Zuia-Gorbeialdea	Dairy	Negative	–	–
20-1004-59	2	Female	Holstein-Friesian	Oiardo	Urkabuztaiz	Zuia-Gorbeialdea	Dairy	Dubious	Untypable	–
20-1004-66	2	Female	Holstein-Friesian	Oiardo	Urkabuztaiz	Zuia-Gorbeialdea	Dairy	**Positive**	**ST10**	–
20-1109-1	2	Female	Mixed breed	Jugo-Zuia	Zuia	Zuia-Gorbeialdea	Beef	Negative	–	–
20-1110-2	2	Female	Pirenaica	Sojoguti	Artziniega	Ayala	Beef	Negative	–	–
20-1110-3	2	Male	Pirenaica	Sojoguti	Artziniega	Ayala	Beef	Negative	–	–
20-1110-4	2	Female	Pirenaica	Sojoguti	Artziniega	Ayala	Beef	Negative	–	–
20-1110-5	2	Female	Pirenaica	Sojoguti	Artziniega	Ayala	Beef	Dubious	Untypable	–
20-1110-7	2	Female	Pirenaica	Sojoguti	Artziniega	Ayala	Beef	Negative	–	–
20-1112-1	2	Female	Holstein-Friesian	Lezama	Amurrio	Ayala	Dairy	Negative	–	–
20-1128-1	2	Female	Holstein-Friesian	Oiardo	Urkabuztaiz	Zuia-Gorbeialdea	Dairy	Negative	–	–
20-1128-3	2	Female	Holstein-Friesian	Oiardo	Urkabuztaiz	Zuia-Gorbeialdea	Dairy	Negative	–	–
20-1138-1	2	Female	Pirenaica	Larrimbe	Amurrio	Ayala	Beef	Negative	–	–
20-1138-2	2	Female	Pirenaica	Larrimbe	Amurrio	Ayala	Beef	Negative	–	–
20-954-1	2	Female	Mixed breed	Jugo	Zuia	Zuia-Gorbeialdea	Beef	Dubious	Untypable	–
20-954-2	2	Female	Mixed breed	Jugo	Zuia	Zuia-Gorbeialdea	Beef	Negative	–	–
20-954-3	2	Female	Mixed breed	Jugo	Zuia	Zuia-Gorbeialdea	Beef	Negative	–	–
20-954-4	2	Female	Mixed breed	Jugo	Zuia	Zuia-Gorbeialdea	Beef	Negative	–	–
20-990-1	2	Female	Holstein-Friesian	Etxabarri	Kuartango	Añana	Dairy	Negative	–	–
20-990-2	2	Female	Holstein-Friesian	Etxabarri	Kuartango	Añana	Dairy	Negative	–	–
20-1000-1	2	Female	Holstein-Friesian	Sojo	Ayala	Ayala	Dairy	Negative	–	–
20-1000-2	2	Female	Holstein-Friesian	Sojo	Ayala	Ayala	Dairy	Negative	–	–
20-1000-3	2	Female	Holstein-Friesian	Sojo	Ayala	Ayala	Dairy	Negative	–	–
20-1000-4	2	Female	Holstein-Friesian	Sojo	Ayala	Ayala	Dairy	Negative	–	–
20-1001-1	2	Female	Holstein-Friesian	Costera	Ayala	Ayala	Dairy	Negative	–	–
20-1002-1	2	Female	Pirenaica	Sojo	Ayala	Ayala	Beef	Dubious	Untypable	–
20-1002-2	2	Female	Pirenaica	Sojo	Ayala	Ayala	Beef	Negative	–	–
19-1895/19	2	Female	Holstein-Friesian	Lukiano	Zuia	Zuia-Gorbeialdea	Dairy	Negative	–	–
19-1143/1	2	Female	Pirenaica	Sojo	Ayala	Ayala	Beef	Negative	–	–
19-1813/1	2	Female	Mixed breed	Jugo	Zuia	Zuia-Gorbeialdea	Beef	Negative	–	–
19-1828/1	2	Female	Mixed breed	Guillarte	Kuartango	Añana	Beef	Negative	–	–
19-1867/1	2	Female	Pirenaica	Oiardo	Urkabuztaiz	Zuia-Gorbeialdea	Beef	Negative	–	–
20-235/1	2	Female	Pirenaica	Oiardo	Urkabuztaiz	Zuia-Gorbeialdea	Beef	Negative	–	–
20-235/1	2	Female	Pirenaica	Oiardo	Urkabuztaiz	Zuia-Gorbeialdea	Beef	Negative	–	–
20-235/2	2	Female	Pirenaica	Oiardo	Urkabuztaiz	Zuia-Gorbeialdea	Beef	Negative	–	–
20-235/3	2	Female	Pirenaica	Oiardo	Urkabuztaiz	Zuia-Gorbeialdea	Beef	Negative	–	–
20-482/1	2	Female	Holstein-Friesian	Oleta	Aramaio	Zuia-Gorbeialdea	Dairy	Negative	–	–
20-482/2	2	Female	Holstein-Friesian	Oleta	Aramaio	Zuia-Gorbeialdea	Dairy	Dubious	Untypable	–
20-489/1	2	Male	Limousin	Izoria	Ayala	Ayala	Beef	Negative	–	–
20-491/1	2	Female	Mixed breed	Aguiñiga	Ayala	Ayala	Beef	Negative	–	–
20-492/2	2	Female	Mixed breed	Salmantón	Ayala	Ayala	Beef	Negative	–	–
20-493/3	2	Female	Limousin	Beotegi	Ayala	Ayala	Beef	Negative	–	–
20-518/1	2	Female	Mixed breed	Salmantón	Ayala	Ayala	Beef	Negative	–	–
20-527/1	2	Female	Holstein-Friesian	Oleta	Aramaio	Zuia-Gorbeialdea	Dairy	Negative	–	–
20-527/2	2	Female	Holstein-Friesian	Oleta	Aramaio	Zuia-Gorbeialdea	Dairy	Negative	–	–
20-538/2	2	Female	Holstein-Friesian	Acosta	Zigoitia	Zuia-Gorbeialdea	Dairy	Dubious	Untypable	–
20-538/3	2	Female	Holstein-Friesian	Acosta	Zigoitia	Zuia-Gorbeialdea	Dairy	Negative	–	–
20-538/4	2	Female	Holstein-Friesian	Acosta	Zigoitia	Zuia-Gorbeialdea	Dairy	Negative	–	–
20-538/5	2	Female	Holstein-Friesian	Acosta	Zigoitia	Zuia-Gorbeialdea	Dairy	Negative	–	–
20-538/6	2	Female	Holstein-Friesian	Acosta	Zigoitia	Zuia-Gorbeialdea	Dairy	Negative	–	–
20-538/7	2	Female	Holstein-Friesian	Acosta	Zigoitia	Zuia-Gorbeialdea	Dairy	Negative	–	–
20-538/8	2	Female	Holstein-Friesian	Acosta	Zigoitia	Zuia-Gorbeialdea	Dairy	Negative	–	–
20-538/9	2	Female	Holstein-Friesian	Acosta	Zigoitia	Zuia-Gorbeialdea	Dairy	Negative	–	–
20-538/10	2	Female	Holstein-Friesian	Acosta	Zigoitia	Zuia-Gorbeialdea	Dairy	Negative	–	–

Next Generation Amplicon Sequencing								*E. bieneusi* ITS-PCR	Sequencing	SNPs ITS-PCR
–	–	–	–	–	–	–	–	Negative	–	–
–	ST10	–	ST21	–	ST24	–	ST26	Negative	–	–
–	–	–	–	–	–	–	–	Negative	–	–
–	ST10	–	ST21	ST23	–	ST25	ST26	Negative	–	–
–	–	–	–	–	–	–	–	Negative	–	–
–	–	–	–	–	–	–	–	Negative	–	–
–	–	–	–	–	–	–	–	Negative	–	–
–	–	–	–	–	–	–	–	Negative	–	–
–	–	–	–	–	–	–	–	Negative	–	–
–	–	–	–	–	–	–	–	Negative	–	–
–	ST10	ST14	ST21	–	–	ST25	ST26	Negative	–	–
–	–	–	–	–	–	–	–	Negative	–	–
–	–	–	–	–	–	–	–	Negative	–	–
–	ST10	ST14	ST21	–	ST24	–	ST26	Negative	–	–
–	–	–	–	–	–	–	–	Negative	–	–
–	–	–	–	–	–	–	–	Negative	–	–
–	–	–	–	–	–	–	–	Negative	–	–
–	–	–	–	–	–	–	–	Negative	–	–
–	–	–	–	–	–	–	–	Negative	–	–
–	–	–	–	–	–	–	–	Negative	–	–
–	–	–	–	–	–	–	–	Negative	–	–
–	ST10	ST14	ST21	ST23	–	ST25	ST26	Negative	–	–
–	ST10	–	–	ST23	–	ST25	ST26	Negative	–	–
–	ST10	ST14	ST21	ST23	–	ST25	ST26	Negative	–	–
–	–	–	–	–	–	ST25	ST26	Negative	–	–
–	ST10	–	–	–	–	ST25	ST26	Negative	–	–
–	–	–	–	–	–	–	–	Negative	–	–
–	–	–	–	–	–	–	–	Negative	–	–
–	–	–	–	–	–	–	–	Negative	–	–
–	–	–	–	–	–	–	–	Negative	–	–
–	ST10	–	–	–	–	ST25	–	Negative	–	–
–	ST10	–	–	–	–	ST25	ST26	Negative	–	–
–	–	–	–	–	–	–	–	Negative	–	–
–	–	–	–	–	–	–	–	Negative	–	–
–	–	–	–	–	–	–	–	Negative	–	–
–	–	–	–	–	–	–	–	Negative	–	–
–	–	–	–	–	–	–	–	Negative	–	–
–	–	–	–	–	–	–	–	Negative	–	–
–	–	–	–	–	–	–	–	Negative	–	–
–	–	–	–	–	–	–	–	Negative	–	–
–	–	–	–	–	–	–	–	Negative	–	–
–	–	–	–	–	–	–	–	Negative	–	–
–	ST10	ST14	ST21	–	ST24	–	ST26	Negative	–	–
–	–	–	–	–	–	–	–	Negative	–	–
–	–	–	–	–	–	–	–	Negative	–	–
–	–	–	–	–	–	–	–	Negative	–	–
–	–	–	–	–	–	–	–	Negative	–	–
–	–	–	–	–	–	–	–	Negative	–	–
–	ST10	–	–	–	–	ST25	ST26	Negative	–	–
–	–	–	–	–	–	–	–	Negative	–	–
–	–	–	–	–	–	–	–	Negative	–	–
–	–	–	–	–	–	–	–	Negative	–	–
–	ST10	–	–	–	–	ST25	ST26	Negative	–	–
–	–	–	–	–	–	–	–	Negative	–	–
–	–	–	–	–	–	–	–	Negative	–	–
–	–	–	–	–	–	–	–	Negative	–	–
–	–	–	–	–	–	–	–	Negative	–	–
–	–	–	–	–	–	–	–	Negative	–	–
–	–	–	–	–	–	–	–	Negative	–	–
–	–	–	–	–	–	–	–	Negative	–	–
–	ST10	–	–	–	–	ST25	ST26	Negative	–	–
–	ST10	–	ST21	ST23	–	ST25	ST26	Negative	–	–
–	ST10	–	–	–	–	ST25	ST26	Negative	–	–
–	–	–	–	–	–	–	–	Negative	–	–
–	ST10	ST14	ST21	–	ST24	–	–	Negative	–	–
–	ST10	–	–	–	–	ST25	ST26	Negative	–	–
–	ST10	–	ST21	ST23	–	ST25	ST26	Negative	–	–
–	ST10	–	ST21	–	–	ST25	ST26	Negative	–	–
–	ST10	ST14	ST21	ST23	–	ST25	ST26	Negative	–	–
–	ST10	–	ST21	ST23	–	–	ST26	Negative	–	–
–	ST10	–	ST21	ST23	–	ST25	ST26	Negative	–	–
–	–	–	–	–	–	–	–	Negative	–	–
–	ST10	–	–	–	–	ST25	ST26	Negative	–	–
–	ST10	–	–	–	–	ST25	ST26	Negative	–	–
–	–	–	–	–	–	–	–	Negative	–	–
–	–	–	–	–	–	–	–	Negative	–	–
–	–	–	–	–	–	–	–	Negative	–	–
–	–	–	–	–	–	–	–	Negative	–	–
–	–	–	–	–	–	–	–	Negative	–	–
–	–	–	–	–	–	–	–	Negative	–	–
–	–	–	–	–	–	–	–	Negative	–	–
–	–	–	–	–	–	–	–	Negative	–	–
–	–	–	–	–	–	–	–	Negative	–	–

–	–	–	–	–	–	–	–	Negative	–	–
–	–	–	–	–	–	–	–	Negative	–	–
–	–	–	–	–	–	–	–	Negative	–	–
–	–	–	–	–	–	–	–	Negative	–	–
–	–	–	–	–	–	–	–	Negative	–	–
–	–	–	–	–	–	–	–	Negative	–	–
–	–	–	–	–	–	–	–	Negative	–	–
ST5	ST10	ST14	–	ST23	–	ST25	ST26	Negative	–	–
–	–	–	–	–	–	–	–	Negative	–	–
–	–	–	–	–	–	–	–	Negative	–	–
–	–	–	–	–	–	–	–	Negative	–	–
–	–	–	–	–	–	–	–	Negative	–	–
–	–	–	–	–	–	–	–	Negative	–	–
–	–	–	–	–	–	–	–	Negative	–	–
–	–	–	–	–	–	–	–	Negative	–	–
–	–	–	–	–	–	–	–	Negative	–	–
–	–	–	–	–	–	–	–	Negative	–	–
–	–	–	–	–	–	–	–	Negative	–	–
–	–	–	–	–	–	–	–	Negative	–	–
–	ST10	ST14	–	–	ST24	–	–	Negative	–	–
–	ST10	ST14	ST21	–	–	ST25	ST26	Negative	–	–
–	ST10	ST14	ST21	ST23	ST24	–	ST26	Negative	–	–
–	ST10	ST14	ST21	ST23	–	ST25	ST26	Negative	–	–
–	ST10	ST14	ST21	ST23	ST24	ST25	ST26	Negative	–	–
–	ST10	ST14	ST21	ST23	–	ST25	ST26	Negative	–	–
–	ST10	–	ST21	ST23	–	ST25	ST26	Negative	–	–
–	ST10	–	ST21	ST23	–	ST25	ST26	Negative	–	–
–	ST10	ST14	ST21	–	–	ST25	ST26	Negative	–	–
–	–	–	–	–	–	–	–	Negative	–	–
–	ST10	ST14	ST21	–	ST24	–	ST26	Negative	–	–
–	–	–	–	–	–	–	–	Negative	–	–
–	ST10	–	ST21	ST23	–	ST25	ST26	Negative	–	–
–	ST10	–	–	–	–	ST25	ST26	Negative	–	–
–	ST10	ST14	ST21	ST23	–	ST25	ST26	Negative	–	–
–	ST10	–	ST21	–	–	ST25	ST26	Negative	–	–
–	ST10	ST14	ST21	ST23	ST24	ST25	–	Negative	–	–
–	ST10	–	ST21	ST23	ST24	ST25	ST26	Negative	–	–
–	–	–	–	–	–	–	–	Negative	–	–
–	–	–	–	–	–	–	–	Negative	–	–
–	ST10	ST14	ST21	–	ST24	ST25	ST26	Negative	–	–
–	ST10	ST14	ST21	ST23	ST24	–	ST26	Negative	–	–
–	–	–	–	–	–	–	–	Negative	–	–
–	ST10	ST14	ST21	–	–	ST25	ST26	Negative	–	–
–	ST10	ST14	ST21	–	–	ST25	ST26	Negative	–	–
–	ST10	ST14	ST21	ST23	–	ST25	ST26	Negative	–	–
–	ST10	ST14	ST21	ST23	–	ST25	ST26	Negative	–	–
–	ST10	–	ST21	–	–	ST25	ST26	Negative	–	–
–	–	–	–	–	–	–	–	Negative	–	–
–	–	–	–	–	–	–	–	Negative	–	–
–	–	–	–	–	–	–	–	Negative	–	–
–	–	–	–	–	–	–	–	Negative	–	–
–	–	–	–	–	–	–	–	Negative	–	–
–	–	–	–	–	–	–	–	Negative	–	–
–	–	–	–	–	–	–	–	Negative	–	–
–	–	–	–	–	–	–	–	Negative	–	–
–	–	–	–	–	–	–	–	Negative	–	–
–	–	–	–	–	–	–	–	Negative	–	–
–	ST10	ST14	ST21	–	–	ST25	ST26	Negative	–	–
–	–	–	–	–	–	–	–	Negative	–	–
–	–	–	–	–	–	–	–	Negative	–	–
–	ST10	–	ST21	ST23	–	ST25	ST26	Negative	–	–
–	ST10	ST14	ST21	ST23	ST24	ST25	ST26	Negative	–	–
–	ST10	ST14	ST21	ST23	–	ST25	ST26	Negative	–	–
–	ST10	ST14	ST21	–	–	ST25	ST26	Negative	–	–
–	ST10	ST14	ST21	–	ST24	ST25	ST26	Negative	–	–
–	ST10	ST14	ST21	–	–	ST25	ST26	Negative	–	–
–	–	–	–	–	–	–	–	Negative	–	–
–	ST10	ST14	ST21	–	–	ST25	ST26	Negative	–	–
–	ST10	ST14	ST21	ST23	ST24	ST25	ST26	Negative	–	–
–	ST10	ST14	ST21	–	ST24	ST25	ST26	Negative	–	–
–	–	–	–	–	–	–	–	Negative	–	–
–	ST10	–	–	–	–	ST25	ST26	Negative	–	–
–	ST10	ST14	ST21	ST23	ST24	ST25	ST26	Negative	–	–
–	ST10	ST14	ST21	ST23	ST24	ST25	ST26	Negative	–	–
–	ST10	ST14	ST21	ST23	ST24	ST25	ST26	Negative	–	–
–	ST10	ST14	ST21	ST23	–	ST25	ST26	Negative	–	–
–	ST10	ST14	ST21	–	–	ST25	ST26	Negative	–	–
–	–	–	–	–	–	–	–	Negative	–	–
–	ST10	ST14	–	–	ST24	–	–	Negative	–	–
–	ST10	ST14	ST21	–	ST24	–	ST26	Negative	–	–
–	ST10	–	–	–	–	ST25	ST26	Negative	–	–
–	–	–	–	–	–	–	–	Negative	–	–
–	ST10	ST14	ST21	–	ST24	ST25	ST26	Negative	–	–
–	ST10	ST14	ST21	–	–	ST25	ST26	Negative	–	–
–	–	–	–	–	–	–	–	Negative	–	–

–	–	–	–	–	–	–	–	Negative	–	–
–	ST10	ST14	–	–	ST24	ST25	ST26	Negative	–	–
–	ST10	ST14	–	–	–	ST25	ST26	Negative	–	–
–	ST10	ST14	–	–	–	ST25	ST26	Negative	–	–
–	ST10	ST14	ST21	ST23	ST24	ST25	ST26	Negative	–	–
–	ST10	ST14	ST21	ST23	ST24	ST25	ST26	Negative	–	–
–	ST10	ST14	ST21	–	–	ST25	ST26	Negative	–	–
–	–	–	–	–	–	–	–	Negative	–	–
–	ST10	ST14	ST21	–	ST24	ST25	ST26	Negative	–	–
–	ST10	ST14	–	–	–	ST25	ST26	Negative	–	–
–	ST10	ST14	–	–	–	ST25	ST26	Negative	–	–
–	–	–	–	–	–	–	–	Negative	–	–
–	–	–	–	–	–	–	–	Negative	–	–
–	–	–	–	–	–	–	–	Negative	–	–
–	–	–	–	–	–	–	–	Negative	–	–
–	–	–	–	–	–	–	–	Negative	–	–
–	–	–	–	–	–	–	–	Negative	–	–
–	–	–	–	–	–	–	–	Negative	–	–
–	–	–	–	–	–	–	–	Negative	–	–
–	–	–	–	–	–	–	–	Negative	–	–
ST5	ST10	ST14	ST21	–	–	ST25	ST26	Negative	–	–
–	ST10	ST14	ST21	–	–	ST25	ST26	Negative	–	–
ST5	ST10	ST14	–	–	–	ST25	ST26	Negative	–	–
–	–	–	–	–	–	–	–	Negative	–	–
–	ST10	ST14	ST21	ST23	ST24	–	ST26	Negative	–	–
–	ST10	–	ST21	–	–	ST25	ST26	Negative	–	–
–	–	–	–	–	–	–	–	Negative	–	–
–	ST10	–	–	–	–	ST25	ST26	Negative	–	–
–	–	–	–	–	–	–	–	Negative	–	–
–	–	–	–	–	–	–	–	Negative	–	–
–	ST10	ST14	ST21	ST23	–	ST25	ST26	Negative	–	–
–	–	–	–	–	–	–	–	Negative	–	–
–	ST10	ST14	ST21	–	–	ST25	ST26	Negative	–	–
–	–	ST14	–	–	–	ST25	ST26	Negative	–	–
–	ST10	ST14	–	–	–	ST25	ST26	Negative	–	–
–	–	–	–	–	–	–	–	Negative	–	–
–	–	–	–	–	–	–	–	Negative	–	–
–	–	–	–	–	–	–	–	Negative	–	–
–	–	–	–	–	–	–	–	Negative	–	–
–	–	–	–	–	–	–	–	Negative	–	–
–	–	–	–	–	–	–	–	Negative	–	–
–	–	–	–	–	–	–	–	Negative	–	–
–	–	–	–	–	–	–	–	Negative	–	–
–	–	–	–	–	–	–	–	Negative	–	–
–	–	–	–	–	–	–	–	Negative	–	–
–	–	–	–	–	–	–	–	Negative	–	–
–	–	–	–	–	–	–	–	Negative	–	–
–	–	–	–	–	–	–	–	Negative	–	–
–	–	–	–	–	–	–	–	Negative	–	–
–	–	–	–	–	–	–	–	Negative	–	–
–	–	–	–	–	–	–	–	Negative	–	–
–	–	–	–	–	–	–	–	Negative	–	–
–	–	–	–	–	–	–	–	Negative	–	–
–	–	–	–	–	–	–	–	Negative	–	–
–	–	–	–	–	–	–	–	Negative	–	–
–	–	–	–	–	–	–	–	Negative	–	–
–	–	–	–	–	–	–	–	Negative	–	–
–	–	–	–	–	–	–	–	Negative	–	–
–	–	–	–	–	–	–	–	Negative	–	–
–	–	–	–	–	–	–	–	Negative	–	–
–	–	–	–	–	–	–	–	Negative	–	–
–	–	–	–	–	–	–	–	Negative	–	–
–	–	–	–	–	–	–	–	Negative	–	–
–	–	–	–	–	–	–	–	Negative	–	–
–	–	–	–	–	–	–	–	Negative	–	–
–	–	–	–	–	–	–	–	Negative	–	–
–	–	–	–	–	–	–	–	Negative	–	–
–	–	–	–	–	–	–	–	Negative	–	–
–	–	–	–	–	–	–	–	Negative	–	–
–	–	–	–	–	–	–	–	Negative	–	–
–	–	–	–	–	–	–	–	Negative	–	–
–	–	–	–	–	–	–	–	Negative	–	–
–	–	–	–	–	–	–	–	Negative	–	–
–	–	–	–	–	–	–	–	Negative	–	–
ST5	ST10	–	–	–	–	ST25	ST26	Negative	–	–
–	ST10	ST14	ST21	–	–	ST25	ST26	Negative	–	–
ST5	ST10	–	–	–	–	ST25	ST26	Negative	–	–
–	–	–	–	–	–	–	–	Negative	–	–
ST5	ST10	–	–	–	–	ST25	ST26	Negative	–	–
ST5	ST10	ST14	ST21	–	–	ST25	ST26	Negative	–	–
ST5	ST10	ST14	ST21	–	–	ST25	ST26	Negative	–	–
–	–	–	–	–	–	–	–	Negative	–	–
–	–	–	–	–	–	–	–	Negative	–	–
–	–	–	–	–	–	–	–	Negative	–	–
–	–	–	–	–	–	–	–	Negative	–	–

–	–	–	–	–	–	–	–	Negative	–	–
–	–	–	–	–	–	–	–	Negative	–	–
–	–	–	–	–	–	–	–	Negative	–	–
–	–	–	–	–	–	–	–	Negative	–	–
–	–	–	–	–	–	–	–	Negative	–	–
–	–	–	–	–	–	–	–	Negative	–	–
ST5	ST10	–	–	–	–	ST25	–	Negative	–	–
ST5	ST10	ST14	–	–	–	ST25	ST26	Negative	–	–
–	–	–	–	–	–	–	–	Negative	–	–
–	–	–	–	–	–	–	–	Negative	–	–
–	–	–	–	–	–	–	–	Negative	–	–
–	–	–	–	–	–	–	–	Negative	–	–
–	–	–	–	–	–	–	–	Negative	–	–
ST5	ST10	ST14	–	–	ST24	–	ST26	Negative	–	–
–	–	–	–	–	–	–	–	Negative	–	–
–	–	–	–	–	–	–	–	Negative	–	–
–	–	–	–	–	–	–	–	**Positive**	**BEB4**	Yes
–	–	–	–	–	–	–	–	Negative	–	–
–	–	–	–	–	–	–	–	Negative	–	–
–	–	–	–	–	–	–	–	Negative	–	–
ST5	ST10	ST14	–	–	–	–	–	Negative	–	–
–	–	–	–	–	–	–	–	Negative	–	–
–	–	–	–	–	–	–	–	Negative	–	–
–	–	–	–	–	–	–	–	Negative	–	–
–	–	–	–	–	–	–	–	Negative	–	–
–	–	–	–	–	–	–	–	Negative	–	–
–	–	–	–	–	–	–	–	Negative	–	–
–	–	–	–	–	–	–	–	Negative	–	–
ST5	ST10	ST14	ST21	–	–	ST25	ST26	Negative	–	–
–	–	–	–	–	–	–	–	Negative	–	–
–	ST10	–	ST21	–	–	–	ST26	Negative	–	–
–	ST10	–	–	–	–	ST25	ST26	Negative	–	–
–	–	–	–	–	–	–	–	Negative	–	–
–	–	–	–	–	–	–	–	Negative	–	–
–	–	–	–	–	–	–	–	Negative	–	–
–	–	–	–	–	–	–	–	Negative	–	–
–	ST10	–	–	ST23	–	ST25	ST26	Negative	–	–
–	–	–	–	–	–	–	–	Negative	–	–
–	–	–	–	–	–	–	–	Negative	–	–
–	–	–	–	–	–	–	–	Negative	–	–
–	–	–	–	–	–	–	–	Negative	–	–
–	–	–	–	–	–	–	–	Negative	–	–
–	–	–	–	–	–	–	–	Negative	–	–
–	ST10	–	–	–	–	ST25	ST26	Negative	–	–
–	–	–	–	–	–	–	–	Negative	–	–
–	–	–	–	–	–	–	–	Negative	–	–
–	–	–	–	–	–	–	–	Negative	–	–
–	–	–	–	–	–	–	–	Negative	–	–
–	–	–	–	–	–	–	–	Negative	–	–
–	–	–	–	–	–	–	–	Negative	–	–
–	–	–	–	–	–	–	–	Negative	–	–
–	–	–	–	–	–	–	–	Negative	–	–
–	–	–	–	–	–	–	–	Negative	–	–
–	–	–	–	–	–	–	–	Negative	–	–
–	ST10	–	–	ST23	–	ST25	ST26	Negative	–	–
–	–	–	–	–	–	–	–	Negative	–	–
–	–	–	–	–	–	–	–	No DNA	–	–
–	–	–	–	–	–	–	–	Negative	–	–
–	–	–	–	–	–	–	–	Negative	–	–
–	–	–	–	–	–	–	–	Negative	–	–
–	–	–	–	–	–	–	–	Negative	–	–
–	–	–	–	–	–	–	–	Negative	–	–
–	–	–	–	–	–	–	–	Negative	–	–
–	–	–	–	–	–	–	–	Negative	–	–
–	–	–	–	–	–	–	–	Negative	–	–
–	–	–	–	–	–	–	–	Negative	–	–
ST5	ST10	ST14	ST21	–	–	ST25	ST26	Negative	–	–
–	–	–	–	–	–	–	–	Negative	–	–
–	–	–	–	–	–	–	–	Negative	–	–
–	–	–	–	–	–	–	–	Negative	–	–
–	–	–	–	–	–	–	–	Negative	–	–
–	–	–	–	–	–	–	–	Negative	–	–
–	–	–	–	–	–	–	–	Negative	–	–
–	–	–	–	–	–	–	–	Negative	–	–
–	ST10	ST14	ST21	ST23	–	ST25	ST26	Negative	–	–
–	–	–	–	–	–	–	–	Negative	–	–
–	–	–	–	–	–	–	–	**Positive**	**BEB4**	Yes
–	–	–	–	–	–	–	–	Negative	–	–
–	–	–	–	–	–	–	–	Negative	–	–
–	–	–	–	–	–	–	–	Negative	–	–
–	–	–	–	–	–	–	–	Negative	–	–
–	–	–	–	–	–	–	–	Negative	–	–
–	–	–	–	–	–	–	–	Negative	–	–
